# Microsatellite stable metastatic colorectal cancer without liver metastasis may be preferred population for regorafenib or fruquintinib plus sintilimab as third-line or above therapy:A real-world study

**DOI:** 10.3389/fonc.2022.917353

**Published:** 2022-09-26

**Authors:** Caiyun Nie, Huifang Lv, Beibei Chen, Weifeng Xu, Jianzheng Wang, Yingjun Liu, Saiqi Wang, Jing Zhao, Yunduan He, Xiaobing Chen

**Affiliations:** ^1^ Department of Medical Oncology, Affiliated Cancer Hospital of Zhengzhou University, Henan Cancer Hospital, Zhengzhou, China; ^2^ State Key Laboratory of Esophageal Cancer Prevention & Treatment, Zhengzhou University, Zhengzhou, China; ^3^ Henan Engineering Research Center of Precision Therapy of Gastrointestinal Cancer, Zhengzhou, China; ^4^ Zhengzhou Key Laboratory for Precision Therapy of Gastrointestinal Cancer, Zhengzhou, China; ^5^ Department of General Surgery, Henan Cancer Hospital, Affiliated Cancer Hospital of Zhengzhou University, Zhengzhou, China

**Keywords:** microsatellite stable, immunotherapy, targeted therapy, liver metastasis, colorectal cancer

## Abstract

**Objectives:**

The antitumor activity of nivolumab plus regorafenib in colorectal cancer from a phase Ib REGONIVO study is encouraging. The present study was conducted to evaluate the efficacy and safety of regorafenib or fruquintinib plus sintilimab as third-line or above therapy in patients with microsatellite stable (MSS) metastatic colorectal cancer.

**Methods:**

Patients with MSS metastatic colorectal cancer who have failed from prior treatment and received regorafenib or fruquintinib plus sintilimab as third-line or above therapy from January 2019 to December 2020 were prospectively analyzed based on real-world clinical practice. The primary end point was progression free survival (PFS). Secondary end points included objective response rate (ORR), disease control rate (DCR), overall survival (OS), and safety.

**Results:**

42 patients received regorafenib plus sintilimab(RS), and the other 30 patients received fruquintinib plus sintilimab(FS). In the general population, the ORR and DCR were 13.9% and 70.8%, and the median PFS and OS was 4.2(95% CI=2.9-5.5) and 10.5 (95% CI=8.6-12.4) months, respectively. There were no statistically significant differences between RS and FS group in PFS (3.5(2.2-4.8) vs. 5.5(3.5-7.5) months, P=0.434) and OS (11.0(7.0-15.0) vs. 10.5(3.8-17.2) months, P=0.486). Subgroup analysis suggested that patients without liver metastasis responded well to this combination regimen (ORR: 21.4% vs. 9.1%) and obtained better OS (26(8.8-43.2) vs. 10.0(7.4-12.6) months, P=0.016). The incidence of Grade 3-4 adverse events (AEs) was 15.3% and the toxicities were generally tolerable and manageable.

**Conclusions:**

Regorafenib or fruquintinib plus sintilimab as third-line or above therapy provide a feasible treatment regimen for MSS metastatic colorectal cancer with tolerated toxicity. Patients without liver metastasis may be the preferred population for this combination regimen.

## Introduction

Colorectal cancer (CRC) is the third most common cancer in men and the second most common cancer in women worldwide (1). In the past, the incidence of colorectal cancer in China was much lower than that in western countries. However, the incidence of colorectal cancer has increased rapidly in recent years, which has become the most common malignant tumor of digestive system. 80% of patients are in the advanced stage when they are diagnosed, which greatly affects the prognosis of colorectal cancer. At present, the level of diagnosis and treatment of metastatic colorectal cancer has made great progress, precision therapy guided by genetic status detection and differentiation of primary tumor sites(left vs. right) has become the main treatment strategy for colorectal cancer (2–4).

Compared with other solid tumors, immunotherapy for colorectal cancer is relatively backward (5). Until 2015, the KEYNOTE-016 study opened the immunotherapy era of microsatellite instability-high(MSI-H) colorectal cancer (6). However, MSI-H tumors account for only about 5%, and the remaining 95% are microsatellite stable(MSS) type colorectal cancer. As a representative of “cold tumors”, immunotherapy seems to be helpless in MSS tumors, and many exploratory studies have failed (7, 8). The antitumor activity of nivolumab plus regorafenib in a colorectal cancer cohort from a phase Ib REGONIVO study is encouraging. The ORR of 25 patients was 36% (ORR of MSS patients was 33%), the median PFS was 7.9 months and the median OS was not reached (9). This is by far the most effective third-line treatment regimen for colorectal cancer. However, this is a phase Ib exploratory study with only 24 patients of MSS colorectal cancer.

Although regorafenib and fruquintinib improved prognosis in metastatic colorectal cancer, the objective response rates of regorafenib and fruquintinib monotherapy in the CORRECT and FRESCO studies were only 1.0% and 4.7%, respectively. In the phase Ib study REGONIVO announced at the 2019 ASCO meeting, the ORR of regorafenib combined with PD-1 antibody was as high as 33% in patients with MSS metastatic colorectal cancer, which were significantly higher than regorafenib and fruquintinib monotherapy. Multi-targeted antiangiogenic TKIs combined with immunotherapy have become a new treatment strategy for MSS colorectal cancer. Since then, a number of prospective single-arm studies explored the efficacy of TKIs combined with immunotherapy in the third-line treatment of MSS metastatic colorectal cancer, including REGONIVO (North America), REGOMUNE, REGOTORI, etc (10–12).

Due to the limited sample size, the results of REGONIVO still need to be further verified. And simultaneously, the efficacy of regorafenib or fruquintinib plus novel immune checkpoint inhibitors (ICIs) has not been reported. The present study was conducted to evaluate the efficacy and safety of regorafenib or fruquintinib plus sintilimab as third-line or above therapy in patients with MSS metastatic colorectal cancer.

## Materials and methods

### Patients population

From January 2019 to December 2020, patients with MSS metastatic colorectal cancer who have failed from prior treatment and received regorafenib or fruquintinib plus sintilimab as third-line or above therapy from Henan Cancer Hospital were prospectively analyzed based on real-world clinical practice. Immunohistochemistry (IHC) staining of four kinds of MMR protein (MLH1,MSH2,MSH6,PMS2) or polymerase chain reaction (PCR) analysis of five microsatellite markers (BAT25,BAT26,D5S346,D2S123,D17S250) were used to determine MSS status of colorectal cancer patients.

### Study treatment

In this study, the patients received regorafenib or fruquintinib in combination with PD-1 inhibitor sintilimab until disease progression, unacceptable toxicity or death. In the regorafenib plus sintilimab group (RS), sintilimab was administered intravenously at a dose of 200 mg once every three weeks, and regorafenib was given orally at a dose of 80 or 120 mg once a day on d1 to d21 every 28 days. In this study, we used regorafenib as the starting dose of 80 mg and adjusted to 120 mg after one week of use, which reduced from 120 to 80 mg in case of intolerable toxicity. In the fruquintinib plus sintilimab group(FS), sintilimab was given as the same dose and fruquintinib was given orally at a dose of 5 mg once a day on d1 to d21 every 4 weeks, which reduced from 4 mg in case of intolerable toxicity.

### Efficacy and safety assessments

After treatment, all patients underwent imaging examination every two cycles (6 weeks) to evaluate the clinical efficacy. The efficacy evaluation criteria are RECIST version 1.1 response evaluation criteria in solid tumors, including complete response (CR), partial response (PR), stable disease (SD), and progressive disease (PD). The objective response rate (ORR) was CR + PR, and the disease control rate (DCR) was CR+ PR and SD. Adverse events (AEs) were assessed according to the Common Terminology Criteria for Adverse Events, version 4.0.

### Statistical analysis

Survival curves of patients were estimated by the Kaplan-Meier method and compared using the log-rank test. The follow-up deadline is January 31, 2022. Progression-free survival (PFS) was defined as starting regorafenib or fruquintinib plus sintilimab as third-line or above treatment to disease progression or death. Overall survival (OS) was defined as the period from the time of regorafenib or fruquintinib plus sintilimab as third-line or above treatment to patient death or last follow-up. Difference between groups were determined by Pearson’s chi squared test or Fisher’s exact test. Receiver operating characteristics (ROC) analysis was applied to determine the cut-off value of Mean Platelet Volume (MPV), Neutrophil-to-Lymphocyte Ratio(NLR), lactate dehydrogenase (LDH) and D-Dimer. Subgroup analysis of predictive factor for PFS and OS was carried out by Cox proportional hazards model. All the statistical descriptive analyses were performed with SPSS 22.0 software (SPSS Inc., IL, US) software. P<0.05 was considered significant.

## Results

### Patient and treatment characteristics

A total of 72 patients with MSS metastatic colorectal cancer who have failed from prior treatment and received regorafenib or fruquintinib plus sintilimab as third-line or above therapy were included in the present study. Patient and treatment characteristics are summarized in **Table 1**. The median age was 57 years (range 32-78), with 36 female patients and 36 male patients. Primary tumor site in 54 patients were left colon, 16 patients had right colon cancer, and the other 2 patients were diagnosed as rectal cancer. Number of metastatic sites in 33(45.8%) patients were 1 or 2, and the other 39(54.2%) patients were 3 or more. The common metastatic sites included lymph node (65.3%), lung (62.5%), liver (61.1%) and peritoneum (27.8%). Regorafenib or fruquintinib plus sintilimab were given as third-line therapy in 39(54.2%) patients, and as fourth-line or above therapy in the other 33(45.8%) patients. All the patients included in the present study were confirmed as MSS status. KRAS, NRAS and BRAF gene were also detected. For KRAS, 32(44.4%) patients were wide type, 32(44.4%) patients were mutant. NRAS and BRAF gene in most patients were wide type(86.1% and 86.1%, respectively). Chemotherapy and targeted therapy are main prior treatment regimen. Chemotherapy regimens include FOLFOX and FOLFIRI. 60(83.3%) patients received anti-VEGF therapy with bevacizumab and 21(29.2%) patients received anti-EGFR therapy with cetuximab. A small proportion of patients(13.9%) had previously received regorafenib, and no patients received fruquintinib in prior therapy. Forty-two patients received regorafenib in combination with sintilimab and the other 30 patients received fruquintinib plus sintilimab. The baseline clinicopathological characteristics in the two groups were similar.

**Table 1:** Patient and treatment characteristics.

Characteristic	Totaln (%)	RP group, n (%)	FP group, n (%)	*P*
Patients, n	72	42	30	—
Age				—
Median	57	59	56	
Range	32-78	35-74	32-78	
Sex				0.339
Female	36 (50.0)	19 (45.2)	17 (56.7)	
Male	36 (50.0)	23 (54.8)	13 (43.3)	
ECOG				0.615
0-1	58 (80.6)	33 (78.6)	25 (83.3)	
2	14 (19.4)	9 (21.4)	5 (16.7)	
Primary tumor site				0.231
Left colon	54 (75.0)	32 (76.2)	22 (73.3)	
Right colon	16 (22.2)	10 (23.8)	6 (20.0)	
Rectum	2 (2.8)	0 (0)	2 (6.7)	
Metastatic site				0.477
Lymph node	47 (65.3)	30 (71.4)	17 (56.7)	
Liver	44 (61.1)	24 (57.1)	20 (66.7)	
Peritoneum	20 (27.8)	9 (21.4)	11 (36.7)	
Lung	45 (62.5)	26 (61.9)	19 (63.3)	
Others	23 (31.9)	16 (38.1)	7 (23.3)	
Number of metastatic sites				0.905
1-2	33 (45.8)	19 (45.2)	14 (46.7)	
≥ 3	39 (54.2)	23 (54.8)	16 (53.3)	
Treatment line				0.719
3	39 (54.2)	22 (52.4)	17 (56.7)	
≥ 4	33 (45.8)	20 (47.6)	13 (43.3)	
MSI status				—
pMMR or MSS	72 (100)	42 (100)	30 (100)	
dMMR or MSI-H	0 (0)	0 (0)	0(0)	
KRAS				0.938
Wide type	32 (44.4)	19 (45.2)	13 (43.3)	
Mutant	32 (44.4)	18 (42.9)	14 (46.7)	
Unknown	8 (11.1)	5 (11.9)	3 (10.0)	
NRAS				0.689
Wide type	62 (86.1)	36 (85.7)	26 (86.7)	
Mutant	1 (1.4)	1 (2.4)	0(0)	
Unknown	9 (12.5)	5 (11.9)	4 (13.3)	
BRAF				0.689
Wide type	62 (86.1)	36 (85.7)	26 (86.7)	
Mutant	1 (1.4)	1 (2.4)	0(0)	
Unknown	9 (12.5)	5 (11.9)	4 (13.3)	
Previous treatment agents				0.647
5-Fluorouracil	68 (94.4)	38 (90.5)	30 (100)	
Oxaliplatin	70 (97.2)	40 (95.2)	30 (100)	
Irinotecan	66 (91.7)	38 (90.5)	28 (93.3)	
Bevacizumab	60 (83.3)	35 (83.3)	25 (83.3)	
Cetuximab	21 (29.2)	14 (33.3)	7 (23.3)	
Regorafenib	10 (13.9)	8 (19.0)	2 (6.7)	
Fruquintinib	0 (0)	0 (0)	0 (0)	

ECOG, Eastern Cooperative Oncology Group performance status; pMMR, mismatch repair proficient; dMMR, mismatch repair deficiency; MSI-H, high microsatellite instability; MSS, microsatellite stable.

### Efficacy

In the general population, CR was not observed, 10 patients achieved PR, 41 patients had SD and 21 patients had PD. The overall ORR and DCR were 13.9% (10/72) and 70.8% (51/72), respectively (**Table 2**). In the RS population, CR was not observed, 5 patients achieved PR, 20 patients had SD and 17 patients had PD. The overall ORR and DCR were 11.9% (5/42) and 59.5% (25/42), respectively. In the FS group, CR was not observed, 5 patients achieved PR, 21 patients had SD and 4 patients had PD. The overall ORR and DCR were 16.7% (5/30) and 86.7% (26/30), respectively. The patients in FS group had higher DCR than RS population (P=0.012), but there was no statistical difference in ORR between the two groups. Meanwhile, the ORR and DCR in patients with different tumor site (left colon vs. right colon), KRAS status (wide type vs. mutant) and metastatic site (with liver metastasis vs. without liver metastasis) were also analyzed, no statistical differences were found between groups. 13.9% of patients have used regorafenib in the previous treatment, there were no statistically significant differences in ORR and DCR between patients with and without prior regorafenib therapy.

**Table 2:** Efficacy of regorafenib or fruquintinib plus sintilimab in metastatic MSS colorectal cancer.

Parameter	Best response				ORR	*P*	DCR	*P*	Median PFS (95%CI)	*P*	Median OS (95%CI)	*P*
	CR	PR	SD	PD								
Total	0	10	41	21	10/72 (13.9)		51/72 (70.8)		4.2 (2.9-5.5)		10.5 (8.6-12.4)	
Treatment programs						0.565		**0.012**		0.434		0.486
RS	0	5	20	17	5/42 (11.9)		25/42 (59.5)		3.5 (2.2-4.8)		11.0 (7.0-15.0)	
FS	0	5	21	4	5/30 (16.7)		26/30 (86.7)		5.5 (3.5-7.5)		10.5 (3.8-17.2)	
Tumor site						0.561		0.264		0.289		0.646
Left colon	0	7	29	18	7/54 (13.0)		36/54 (66.7)		3.6 (2.5-4.7)		11.0 (8.9-13.1)	
Right colon	0	3	10	3	3/16 (18.8)		13/16 (81.3)		7.0 (1.1-12.9)		10.0 (4.7-15.3)	
KRAS status						0.281		0.777		0.784		0.665
Wide type	0	6	18	8	6/32 (18.8)		24/32 (75.0)		4.5 (3.2-5.4)		11.0 (8.0-14.0)	
Mutant	0	3	20	9	3/32 (9.4)		23/32 (71.9)		5.0 (2.9-7.1)		10.5 (7.4-13.6)	
Metastatic site						0.140		0.658		0.075		**0.016**
Liver	0	4	28	12	4/44 (9.1)		32/44 (72.7)		3.5 (2.4-4.6)		10.0 (7.4-12.6)	
Without liver	0	6	13	9	6/28 (21.4)		19/28 (67.9)		4.5 (1.5-7.5)		26.0 (8.8-43.2)	
Prior R therapy						0.338		0.713		0.483		0.213
Yes	0	0	8	2	0		8/10 (80.0)		3.6 (2.5-4.7)		10.0 (5.9-14.1)	
No	0	10	33	19	10/62 (16.1)		43/62 (69.4)		4.3 (2.6-6.0)		11.3 (7.9-14.7)	

CR, complete response; PR, partial response; SD, stable disease; PD, progressive disease; ORR, overall response rate; DCR, disease control rate; PFS, progression free survival; OS, overall survival. Bold values: P<0.05.

**Figure 1:**
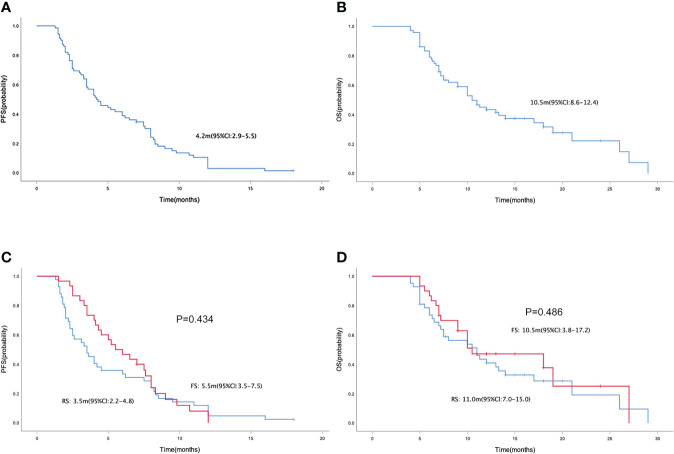
Kaplan-Meier curve of PFS **(A)** and OS **(B)** in the general population. Kaplan-Meier curve of PFS **(C)** and OS **(D)** in the regorafenib plus sintilimab(RS), and fruquintinib plus sintilimab(FS) group.

**Figure 2:**
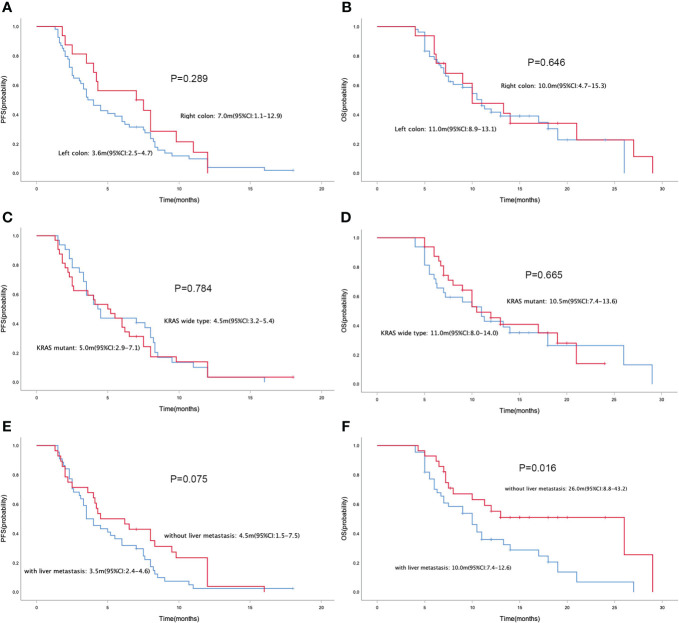
Kaplan-Meier curve of PFS **(A)** and OS **(B)** in patients with different primary tumor site (left colon vs. right colon). Kaplan-Meier curve of PFS **(C)** and OS **(D)** in patients with different KRAS status (wide type vs. mutant). Kaplan-Meier curve of PFS **(E)** and OS **(F)** in patients with liver metastasis or without liver metastasis.

In the general population, the median PFS and median OS were 4.2 (95% CI= 2.9-5.5) and 10.5 (95% CI= 8.6-12.4) months, respectively (**Figures 1A, B**). The median PFS were 3.5 (95% CI= 2.2-4.8) and 5.5 (95% CI= 3.5-7.5) months in the RS and FS population, respectively (P = 0.434; **Figure 1C**). The median OS in the two groups were 11.0 (95% CI=7.0-15.0) months and 10.5 (95% CI=3.8-17.2) months, respectively (P = 0.486; **Figure 1D**). Simultaneously, the median PFS and OS in patients with different tumor site (left colon vs. right colon), KRAS status (wide type vs. mutant), metastatic site (with liver metastasis vs. without liver metastasis) and prior regorafenib therapy (Yes vs. No) were also compared, no statistical differences were found between groups with different tumor site, KRAS status and with or without prior regorafenib therapy (**Figure 2**). However, although no statistical difference exists in median PFS between patients with liver metastasis or without liver metastasis (3.5(2.4-4.6) vs. 4.5(1.5-7.5) months, P=0.075), the median OS in patients without liver metastasis was significantly better than patients with liver metastasis (26.0(8.8-43.2) vs. 10.0(7.4-12.6) months, P=0.016, **Figure 2**).

### Subgroup analysis of predictive factors

**Figure 3:**
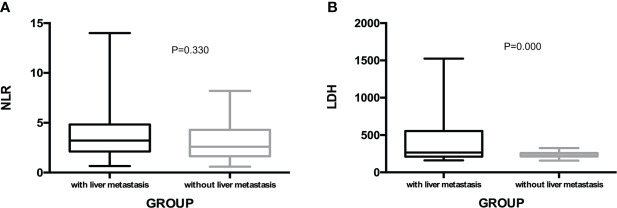
NLR **(A)** and LDH **(B)** level in colorectal cancer patients with and without liver metastasis.

The present study also performed univariate analysis to evaluate the predictive value of clinicopathologic factors for PFS and OS, including sex (male vs. female), age (<65 vs. ≥65), treatment program (RS vs. FS), primary tumor site (left colon vs. right colon), liver metastasis (with vs. without), KRAS status (wide type vs. mutant), MPV (<9.9 vs. ≥9.9), NLR (<2.15 vs. ≥2.15), LDH (<312 vs. ≥312) and D-Dimer (<0.89 vs. ≥0.89). None of the above factors were found to be predictive factors for PFS. For OS, only with or without liver metastasis was confirmed to be a potential predictive factor (P=0.021, **Table 3**). We compared NLR and LDH between colorectal cancer patients with and without liver metastasis and no statistical difference was found in NLR between the two groups (P=0.330). However, the baseline LDH levels in patients with liver metastasis were significantly higher than those without liver metastasis (median level: 278 U/L vs. 218 U/L, P=0.000, **Figure 3**).

**Table 3:** Exploratory univariate analysis of factors to predict PFS and OS.

Parameter	No. of patients(%)	Univariate analysis for PFS			Univariate analysis for OS		
		HR	95% CI	*P*	HR	95% CI	*P*
Sex		1.023	0.635-1.647	0.926	1.044	0.587-1.854	0.844
Male	36 (50.0)						
Female	36 (50.0)						
Age		0.592	0.335-1.048	0.072	0.679	0.345-1.335	0.262
<65	53 (73.6)						
≥65	19 (26.4)						
Treatment program		0.829	0.509-1.351	0.452	0.815	0.453-1.464	0.493
RS	42 (58.3)						
FS	30 (41.7)						
Primary tumor site		0.742	0.417-1.322	0.311	0.852	0.426-1.704	0.651
Left colon	54 (75.0)						
Right colon	16 (22.2)						
Liver metastasis		0.642	0.387-1.065	0.086	0.478	0.255-0.894	**0.021**
Yes	44 (61.1)						
No	28 (38.9)						
KRAS		1.070	0.646-1.772	0.794	0.875	0.473-1.620	0.671
Wide type	32 (44.4)						
Mutant	32 (44.4)						
MPV		1.144	0.703-1.861	0.589	0.808	0.451-1.446	0.473
<9.9	43 (59.7)						
≥9.9	29 (40.3)						
NLR		0.933	0.550-1.584	0.798	1.662	0.855-3.231	0.134
<2.15	21 (29.2)						
≥2.15	51 (70.8)						
LDH		1.332	0.760-2.333	0.316	1.698	0.885-3.257	0.111
<312	55 (76.4)						
≥312	17 (23.6)						
D-Dimer		1.320	0.809-2.154	0.267	1.288	0.726-2.287	0.387
<0.89	45 (62.5)						
≥0.89	27 (37.5)						

MPV, Mean Platelet Volume; NLR, Neutrophil-to-Lymphocyte Ratio; LDH, lactate dehydrogenase. Bold values: P<0.05.

### Safety

Most of the adverse events were grade 1-2 in severity and the incidence of Grade 3-4 AEs was 15.3% (**Table 4**). No unexpected side effects or treatment-related death were observed. The dose reduction and treatment interruptions as a result of serious adverse events occurred in 28 (38.9%) and 33 (45.8%) patients, respectively. The most common treatment-related hematological AEs were increased ALT/AST (n=13, 18.1%), anemia (n=11, 15.3%), decreased white blood count (n=6, 8.3%), hyperbilirubinemia (n=5,6.9%), and decreased platelet (n=4, 5.6%). Non-hematological treatment-related AEs were fatigue (n=23, 31.9%), decreased appetite (n=22, 30.6%), secondary hypertension (n=17, 23.6%), hypothyroidism (n=15, 20.8%), oral mucositis (n=14, 19.4%), diarrhea (n=10, 13.9%), hand-foot syndrome (n=8, 11.1%), proteinuria (n=4, 5.6%), rash (n=4, 5.6%), pneumonitis (n=3, 4.2%), pyrexia (n=2, 2.8%). Grade 3-4 AEs were decreased platelet (n=1, 1.4%), increased ALT/AST (n=2, 2.8%), secondary hypertension (n=4, 5.6%), hand-foot syndrome (n=2, 2.8%), rash (n=1, 1.4%) and pneumonitis (n=1, 1.4%).

**Table 4:** Treatment-related adverse events (TRAEs).

Adverse Event	All grade N, %	≥ Grade3 N, %
Hematologic		
Decreased white blood count	6 (8.3)	0 (0)
Anemia	11 (15.3)	0 (0)
Decreased platelet	4 (5.6)	1 (1.4)
Increased ALT/AST	13 (18.1)	2 (2.8)
Hyperbilirubinemia	5 (6.9)	0 (0)
Non- Hematologic		
Fatigue	23 (31.9)	0 (0)
Decreased appetite	22 (30.6)	0 (0)
Oral mucositis	14 (19.4)	0 (0)
Diarrhea	10 (13.9)	0 (0)
Secondary hypertension	17 (23.6)	4 (5.6)
Hand-foot syndrome	8 (11.1)	2 (2.8)
Proteinuria	4 (5.6)	0 (0)
Rash	4 (5.6)	1 (1.4)
Pneumonitis	3 (4.2)	1 (1.4)
Pyrexia	2 (2.8)	0 (0)
Hypothyroidism	15 (20.8)	0

## Discussion

Colorectal cancer is a highly heterogeneous disease, and stratified therapy based on genetic testing is currently the main strategy for third-line treatment of metastatic colorectal cancer. For MSI-H colorectal cancer, immunotherapy is preferred recommended treatment regimen (13–15). However, for the vast majority of patients with MSS type, single-agent chemotherapy and immunotherapy are almost ineffective. The international multicenter phase 3 clinical CORRECT study for the first time confirmed the OS benefit of regorafenib in refractory advanced colorectal cancer. The results showed that the median OS of the regorafenib group reached 6.4 months, which was significantly longer than that of the placebo control group (16). The FRESCO study evaluated the efficacy and safety of fruquintinib as third-line or later therapy in 416 patients with metastatic CRC. The results showed that the median OS of patients in the fruquintinib group was 9.3 months, which was 2.7 months longer than that in the placebo group, and the median PFS was extended from 1.8 months in the placebo group to 3.7 months (17). Based on the above clinical trials, small molecule tyrosine kinase inhibitors including regorafenib and fruquintinib are the standard third-line treatment options for colorectal cancer recommended in the current guidelines.

In addition to monotherapy, regorafenib and fruquintinib combined with immunotherapy has become a new treatment strategy. Most studies obtained consistent findings with the REGONIVO study, however in REGONIVO (North America) trial, the ORR of regorafenib combined with nivolumab was 7% in patients with MSS metastatic colorectal cancer, PFS and OS were 1.8 and 11.9 months respectively, which were worse than previous studies. Therefore, the ideal drug selection, dosage, benefit population for TKIs combined with immunotherapy in metastatic colorectal cancer still need to be further explored. Our present study evaluated the efficacy of regorafenib and fruquintinib plus sintilimab as third-line or above therapy in patients with MSS metastatic colorectal cancer, the overall ORR and DCR reached 13.9% and 70.8%, respectively. Although the ORR in our study is lower than previous clinical trials, it is worth noting that 54.2% patients received regorafenib or fruquintinib plus sintilimab as third-line therapy, and as fourth-line or above therapy in the other 45.8% patients. At the same time, the DCR was 70.8%, and median PFS and OS reached 4.2 and 10.5 months respectively, so regorafenib or fruquintinib plus sintilimab therapy still achieved a good therapeutic effect in such a relatively late-line patient population.

MSS-type colorectal cancer has been referred to as a “cold tumor” due to the low response to single-agent immunotherapy. Combination immunotherapy, including chemotherapy, targeted therapy or other immunomodulatory agents, to change it from “cold tumor” to “hot tumor”, is being actively explored. Multi-targeted antiangiogenic TKIs, including regorafenib and fruquintinib achieved better effect. Immunosuppressive cells such as regulatory T cells (Tregs) and tumor associated macrophages (TAMs) exist in the tumor microenvironment of patients with MSS colorectal cancer, which can suppress T cell activity (18–20). Basic research has shown that regorafenib can relieve the immunosuppression of Treg and TAM cells on T cells by inhibiting CSF1R and VEGFR to enhance the efficacy of immunotherapy (21, 22). Several previous retrospective studies have compared the efficacy of regorafenib and fruquintinib in combination with immunotherapy, and the results are inconsistent (23, 24). In our present study, except for DCR, fruquintinib was superior to regorafenib(86.7% vs. 59.5%, P=0.012), there were no significant differences in ORR, PFS and OS between the two groups.

However, not all MSS colorectal cancer patients respond well to this combination therapy mode, which means that it is necessary to further explore effective biomarkers and stratify the patient population to improve the survival benefit of patients. Subgroup analysis of predictive factors for PFS and OS demonstrated that the clinical benefit of this regimen was not related with sex, age, treatment program, primary tumor site, KRAS status, MPV, NLR, LDH and D-Dimer. However, patients without liver metastasis responded well to this combination regimen(ORR: 21.4% vs. 9.1%), and meantime although no statistical difference exists in median PFS between patients with liver metastasis or without liver metastasis, the median OS in patients without liver metastasis was significantly better than patients with liver metastasis. Liver is a common metastatic site of colorectal cancer, and liver metastasis is also the main cause of death in patients with colorectal cancer. Metastasis site may be a predictor of immunotherapy efficacy (25). In colorectal cancer, patients with liver metastasis have a suboptimal response to immunotherapy and have a poor prognosis (26). The REGOTORI study evaluated the efficacy of regorafenib plus toripalimab in patients with metastatic colorectal cancer, the ORR of patients with liver metastases was lower than that of patients without liver metastases (8.7% and 30.0%, respectively). Our present study yielded consistent findings that patients without liver metastasis responded well to this combination regimen and benefited more. Recent studies have shown that liver metastases suppress systemic antitumor immune responses and suppress immunotherapy efficacy by reducing systemic CD8+ T cells (27). LDH might be an indirect sign of activated tumor angiogenesis and immunosuppression, and our study also found that patients with liver metastasis had higher levels of LDH. For MSS metastatic colorectal cancer patients with liver metastasis, it is necessary to explore more effective treatment options.

13.9% of patients have used regorafenib in the previous treatment, there were no statistically significant differences in ORR, DCR, median PFS and median OS between patients with prior regorafenib therapy and without prior regorafenib therapy. This suggests that regorafenib or fruquintinib plus sintilimab remains an optional treatment strategy for patients who have failed previous regorafenib therapy.

The toxicity profile of this combination regimen was tolerable and was comparable with previous studies (28–30). The REGONIVO study demonstrated that combination of regorafenib 80 mg plus nivolumab had a manageable safety profile and encouraging antitumor activity. In this study, the dose reduction as a result of serious adverse events occurred in 28 (38.9%) patients. With this dose adjustment strategy, the treatment was well tolerated in patients. Our study has several strengths and limitations, because it is an observational study and the number of patients included is not large. Future validation clinical trials would be needed to confirm the value of regorafenib or fruquintinib plus sintilimab as third-line or above therapy in MSS metastatic colorectal cancer.

## Conclusion

In conclusion, these data confirm that regorafenib or fruquintinib plus sintilimab as third-line or above therapy provide a feasible treatment regimen for MSS metastatic colorectal cancer with tolerated toxicity. Patients without liver metastases may be the preferred population for this combination regimen.

## Data availability statement

The original contributions presented in the study are included in the article/supplementary material. Further inquiries can be directed to the corresponding author.

## Ethics statement

The studies involving human participants were reviewed and approved by This study was carried out in accordance with the ethical guidelines of the 1975 Declaration of Helsinki and was approved by the ethics committee of the Affiliated Cancer Hospital of Zhengzhou University (KY-0192). The patients/participants provided their written informed consent to participate in this study.

## Author contributions

CN and XC designed the research, analyzed the data and drafted the paper. CN, HL, BC, WX, JW and YL were mainly responsible for data collection and analysis. CN, SW, JZ and YH were primarily responsible for statistical analysis. All authors contributed to the article and approved the submitted version.

## Funding

This work was supported by Medical Science and Technique Foundation of Henan Province (No. 212102310623), Medical Science and Technique Foundation of Henan Province (No. SB201901101), Young and Middle-aged Health and Technology Innovation Leading Talent Project of Henan Province (No. YXKC2020008), 1000 Talents Program of Central plains (No. 204200510023) and the Sate Key Laboratory of Esophageal Cancer Prevention & Treatment (No. Z2020000X).

## Conflict of interest

The authors declare that the research was conducted in the absence of any commercial or financial relationships that could be construed as a potential conflict of interest.

## Publisher’s note

All claims expressed in this article are solely those of the authors and do not necessarily represent those of their affiliated organizations, or those of the publisher, the editors and the reviewers. Any product that may be evaluated in this article, or claim that may be made by its manufacturer, is not guaranteed or endorsed by the publisher.
